# Growth and Product Formation of *Clostridium ljungdahlii* in Presence of Cyanide

**DOI:** 10.3389/fmicb.2018.01213

**Published:** 2018-06-13

**Authors:** Florian Oswald, Michaela Zwick, Ola Omar, Ernst N. Hotz, Anke Neumann

**Affiliations:** Institute of Process Engineering in Life Sciences, Section II, Technical Biology, Karlsruhe Institute of Technology, Karlsruhe, Germany

**Keywords:** cyanide, *Clostridium ljungdahlii*, crude syngas, ethanol formation, syngas fermentation, syngas impurities, growth inhibition

## Abstract

Cyanide is a minor constituent of crude syngas whose content depends on the feedstock and gasification procedure. It is a known poison to metal catalysts and inhibits iron-containing enzymes like carbon monoxide dehydrogenase of acetogenic organisms. Therefore, it is considered a component that has to be removed from the gas stream prior to use in chemical synthesis or syngas fermentation. We show that the growth rate and maximum biomass concentration of *Clostridium ljungdahlii* are unaffected by cyanide at concentrations of up to 1.0 mM with fructose as a carbon source and up to 0.1 mM with syngas as a carbon source. After the culture is adapted to cyanide it shows no growth inhibition. While the difference in growth is an increasing lag-phase with increasing cyanide concentrations, the product spectrum shifts from 97% acetic acid and 3% ethanol at 0 mM cyanide to 20% acetic acid and 80% ethanol at 1.0 mM cyanide for cultures growing on (fructose) and 80% acetic acid and 20% ethanol at 0.1 mM cyanide (syngas).

## Introduction

Hydrogen, carbon monoxide, and carbon dioxide are the main constituents of gas yielded by gasification of coal or biomass, also called syngas. Gasification is a thermal process that degrades carbon and hydrogen containing feedstocks into their molecular building blocks. The gas composition varies depending on gasifier type, feedstock, and process mode but consists mainly of CO, CO_2_, H_2_, and N_2_, if air is used as the gasification medium. In addition to these main constituents, crude syngas also contains variable amounts of methane (CH_4_) and C_2_-compounds such as ethane (C_2_H_6_), ethylene (C_2_H_4_), and acetylene (C_2_H_2_); tar components like benzene, toluene, xylene, and naphthalene; halogens such as hydrogen chloride (HCl) and hydrogen fluoride (HF); sulfur compounds like hydrogen sulfide (H_2_S), carbonyl sulfide (COS), and carbonyl disulfide (CS_2_); and nitrogen species such as nitrogen oxides (NO_x_), ammonia (NH_3_), and hydrogen cyanide (HCN) as well as oxygen (O_2_) and reactive oxygen species (ROS) (Hofbauer et al., 2009). The amounts of these minor constituents vary with feedstock and gasification method. Cyanide levels of crude syngas, for example, range from below 25 (Boerrighter et al., 2013) to 2500 ppm (Broer et al., 2015).

The minor constituents of syngas are known catalyst poisons for chemical applications and need to be removed. Compared to the chemical route, biological syngas conversion uses, for example, acetogenic microorganisms, which catalyze the conversion of syngas to acetic acid or ethanol at ambient temperature and pressures. Some of the named minor constituents of syngas like NH_3_ are usable substrates for these microorganisms. Since gas conditioning makes for about 22% of the total investment costs of biomass to liquid (BTL) plants and one-third of the investment costs for gasification alone (Hannula and Kurkela, 2013), processes capable of using crude syngas offer a financial advantage over other processes that depend on purified syngas. If crude syngas is supposed to be used for syngas fermentation, understanding of the effects of syngas impurities is necessary to avoid delays in reaching full-scale production capacity and additional costs (Lane, 2014).

Two important enzymes in the metabolism of acetogens that are affected by gas impurities are carbon monoxide dehydrogenase (CODH) and hydrogenase. CODHs are found in various organisms and some inhibitory effects are well described in the literature as reviewed below. Among the above mentioned impurities cyanide is a common inhibitor of CODH. Thauer et al. (1974) show that carbon monoxide oxidation activity of cell-free extracts of *Clostridium pasteurianum* is inhibited by cyanide at a concentration of 10 μM and Ha et al. (2007) find that 75 μM cyanide completely inactivates CODH from *Carboxydothermus hydrogenoformans*. CODH inhibition by cyanide is, however, reversed by CO and the rate of inactivation has been reported to be reduced by 100-fold in the presence of CO at 10 μM (Thauer et al., 1974). Other researchers have described the same competitive behavior of CODH inhibition by cyanide for *Methanosarcina thermophila* (Terlesky et al., 1986), *Methanosarcina barkeri* (Grahame and Stadtman, 1987), *Moorella thermoacetica* (formerly *Clostridium thermoaceticum*) (Diekert and Thauer, 1978; Ragsdale et al., 1983a; Anderson et al., 1993), *Clostridium formicoaceticum* (Diekert and Thauer, 1978), *Rhodospirillum rubrum* (Ensing et al., 1989; Smith et al., 1992), *Acetobacterium woodii* (Ragsdale et al., 1983b), and *C. hydrogenoformans* (Ha et al., 2007). The competitive behavior and the protection against cyanide inhibition by CO indicate a common binding site for CN^-^ and CO (Terlesky et al., 1986; Grahame and Stadtman, 1987; Ensing et al., 1989). Anderson et al. (1993) discover that CN^-^ binds directly to the C-cluster of *M. thermoacetica* and Ha et al. (2007) show that cyanide competes with CO at the (Ni–4Fe–5S)-cluster of CODH from *C. hydrogenoformans*. It is suggested that the C-cluster is part of the active site in all Ni–Fe CODHs (Anderson et al., 1993). Cell-free extracts of *C. pasteurianum* regained CODH activity even without carbon monoxide treatment and Thauer et al. (1974) measure the activity for rhodanese reaction that detoxifies cyanide and converts it to thiocyanate and sulfite.

Sulfur components are another class of CODH inhibitors and of those COS and hydrogen sulfide are of interest (Hyman et al., 1989; Vega et al., 1990). Total inhibition of CO oxidation activity of purified CODH from *R. rubrum* is completed in less than 5 s and hints toward a rapid-equilibrium inhibition. The inhibition kinetic seems to follow a ping-pong mechanism and CODH has to be in an oxidized state before COS can bind and the whole inhibition process is more complex than with cyanide. Nevertheless, the inhibition is fully reversible when COS is removed from the atmosphere and CODH is incubated in the presence of CO (Hyman et al., 1989). Experiments by Hyman et al. (1989) revealed that in the presence of 11.5 μM COS in solution the inhibitory effect of 100 μM cyanide decreased by a factor of 5, indicating that COS, CN^-^, and CO share the same binding site. With increasing aqueous COS concentration the protective effect against cyanide inhibition saturates at 70% remaining activity (250 μM COS). Although COS, SO_2_, and CS_2_ are able to reverse cyanide inhibition, the latter two have proved ineffective as inhibitors for CO oxidation (Hyman et al., 1989). Vega et al. (1990) investigate four microorganisms on their tolerance against H_2_S and COS components in syngas containing 39.5% of each compound. *R. rubrum* shows no changes in growth and substrate consumption in the presence of H_2_S in the gaseous phase up to 26.3% but the growth is inhibited at a COS gaseous concentration of 6.6%. CO utilization by *Peptostreptococcus productus* is inhibited at concentrations higher than 19.7% of COS and H_2_S, respectively. *M. barkeri* remains unaffected by H_2_S and COS up to concentrations of 26.3% whereas *Methanobacterium formicum* shows significant inhibition at 13.2% H_2_S and 6.6% COS (Vega et al., 1990). *Clostridium ljungdahlii* cultures previously grown in Na_2_S containing media showed no effects on growth or CO consumption at H_2_S or COS concentrations in the gaseous phase of up to 5.2% but at 9.9% both growth and CO consumption essentially stopped (Klasson et al., 1992).

Hyman and Arp (1988) published a review article about the inhibitory effects of acetylene on metalloenzymes and pointed out that studies on acetylene inhibition of purified hydrogenases have only focused on enzymes of aerobic, nitrogen-fixing microorganisms. These organisms have dimeric, Ni-containing hydrogenases. Also hydrogenase of the aerobic proteobacterium *Azotobacter vinelandii* is insensitive to acetylene in the presence of trace amounts of hydrogen. Hydrogenases are insensitive to cyanide but are inhibited by carbon monoxide (Hyman and Arp, 1988). Isolated Ni–Fe hydrogenase from *Desulfovibrio gigas* retains less than 20% of their activity in the presence of 10% acetylene in the gaseous phase without hydrogen. The same study states that acetylene has no inhibitory effect on Fe-only hydrogenase from *D. vulgaris* and Ni–Fe–Se hydrogenase from *D. baculatus* retains 50% activity when incubated with 100% acetylene. Selenium (Se) containing Ni–Fe hydrogenases are less sensitive to acetylene than Ni–Fe hydrogenases without Se. The same tendency can be found for Ni–Fe and Ni–Fe–Se hydrogenases from *Methanococcus voltae* and *M. thermophila* (He et al., 1989). Experiments with whole cells of *R. rubrum* revealed also an insensitivity of hydrogen consumption in the presence of acetylene (Maness and Weaver, 2001).

Another inhibitor of hydrogenase activity is nitric oxide (NO). In cell-free extracts of *Proteus vulgaris* 87% hydrogenase activity is lost when exposed to 20 ppm NO in the gas phase (Krasna and Rittenberg, 1954). It has been reported that the growth of *Clostridium carboxidivorans* is inhibited when exposed to 130 ppm NO after inoculation but continued growth is observed when 130 ppm NO is applied after the cells reached the first stationary growth phase (Ahmed and Lewis, 2006). 40 ppm NO show no inhibitory effects even when applied straight after inoculation. Studies on whole-cell hydrogenase activity of *C. carboxidivorans* show that the activity decreases at NO concentrations higher than 40 ppm with 10–95% inhibition between 60 and 130 ppm. Hydrogenase inhibition by NO is non-competitive (Ahmed and Lewis, 2006).

In literature about the usage of syngas or waste gases for syngas fermentation, the removal of gas impurities is named as a crucial point. As can be seen from the mentioned literature, studies of effects of syngas impurities usually focus on isolated enzymes or cell extracts. So far only few reports can be found in literature about the effects of syngas impurities on growing acetogens. To our knowledge, no reports about the effects of cyanide on growing acetogens exist. Therefore, this work investigates the effect of cyanide ions on growth and product formation of *C. ljungdahlii* under heterotrophic and autotrophic conditions.

## Materials and Methods

If not stated differently, all chemicals were purchased from Carl-Roth (Germany). The strain used in this work was *C. ljungdahlii* DSM13528. Medium for batch cultivation of *C. ljungdahlii* in flasks was based on Tanner (2007) and contained per liter: 20 g 2-(*N*-morpholino)ethanesulfonic acid (MES), 0.5 g yeast extract (BD, United States), 2 g NaCl, 0.33 g NH_4_Cl, 0.25 g KCl, 0.25 g KH_2_PO_4_, 0.5 g MgSO_4_⋅7H_2_O, 0.1 g CaCl_2_⋅2H_2_O, 10 mL trace element solution (composition see below), 10 mL vitamin solution (composition see below), and 0.001 g resazurin. Trace element solution contained per liter: 2 g nitrilotriacetic acid, 1 g MnSO_4_⋅H_2_O, 0.567 g FeSO_4_⋅7H_2_O, 0.2 g CoCl_2_⋅6H_2_O (Riedel-de Haën, Germany), 0.2 g ZnSO_4_⋅7H_2_O, 0.02 g CuCl_2_⋅2H_2_O, 0.02 g NiCl_2_⋅6H_2_O, 0.02 g Na_2_MoO_4_⋅2H_2_O, 0.02 g Na_2_SeO_3_⋅5H_2_O, and 0.022 g Na_2_WO_4_⋅2H_2_O. Vitamin solution contained per liter: 0.002 g biotin, 0.002 g folic acid, 0.01 g pyridoxine (Alfa Aesar, Germany), 0.005 g thiamine-HCl, 0.005 g riboflavin, 0.005 g niacin, 0.005 g Ca-pantothenate, 0.005 g cobalamin, 0.005 g 4-aminobenzoic acid, and 0.005 g lipoic acid (Cayman Chemical, United States). The medium was prepared using strict anaerobic techniques and the pH was adjusted to 5.9 using KOH before bottling. Bottles were made anaerobic using a gas mixture containing 20 vol% carbon dioxide in nitrogen (Air Liquide, France). After autoclaving at 121°C, 1 g cystein–HCl⋅H_2_O per liter was added.

**FIGURE 1:**
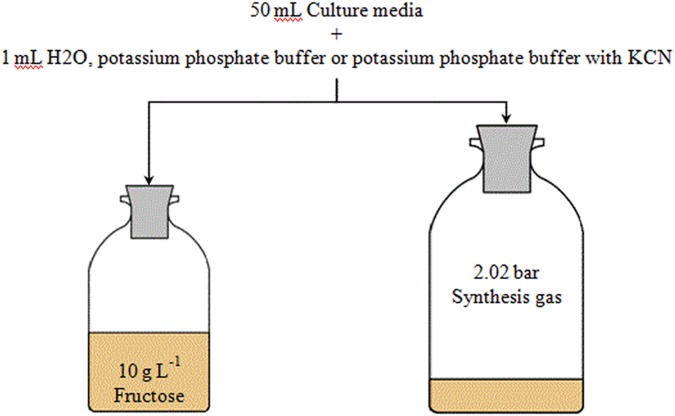
Overview of cyanide experiments with *C. ljungdahlii*. Effects of cyanide are investigated in both fructose and syngas growing cultures with cyanide concentrations of 0, 0.025, 0.05, 0.1, and 1.0 mmol L^-1^. Experiments are conducted in triplicates.

Due to the toxicity of cyanide, it was added in liquid form. Cyanide was prepared as anaerobic potassium cyanide (KCN) solution in 100 mM potassium phosphate buffer at pH 11 to prevent hydrogenation of CN^-^ and degassing of HCN during sterilization and storage. All buffers were prepared in sealed serum bottles. *C. ljungdahlii* DSM13528 was grown in 50 mL complex media with either 10 g L^-1^ fructose or 2.02 bar absolute pressure of synthesis gas. The gas atmosphere from anaerobic and autoclaved bottles was replaced with syngas prior to inoculation of syngas experiments by evacuation of the bottles and pressurization with syngas. Fructose experiments were conducted in 125 mL serum bottles and syngas experiments in 250 mL serum bottles. **Figure 1** gives an overview of the experiments. The following final cyanide concentrations were investigated (all numbers are in millimolar): 0 (only phosphate buffer is added), 0.025, 0.05, 0.1, and 1.0. To achieve the desired KCN concentrations for every flask 1 mL of potassium phosphate buffer with or without cyanide was added per 50 mL of culture media. Cultures with 1 mL sterile anaerobic water per 50 mL of culture media were used as controls for growth and product formation. All experiments were conducted in triplicates. Cultures were cultivated at 37°C without shaking. Headspace pressure in cultures growing with syngas was used as an indicator of substrate consumption and was measured before and after collection of liquid samples as well as before and after collection of gas samples using a digital manometer (Greisinger-electronic GmbH, Germany). The sample size for liquid samples was 2 and 5 mL for gaseous samples. Gas samples were analyzed with a 3000 micro-GC (INFICON, Switzerland) to determine the consumption of the individual components of the syngas used. The micro-GC was equipped with one 10 m molsieve module for CO, H_2_, and N_2_ detection and a 10 m PoraPlot Q module for CO_2_ detection. All modules were equipped with a thermal conductivity detector and analytics used isothermal conditions at 80°C.

Cell concentrations were determined using an Ultrospec1100pro spectrophotometer (Amersham Bioscience, United States) at a wavelength of 600 nm. The optical density (OD) of 1 mL of a liquid sample was measured, then cells were removed via centrifugation at 16,100 ×*g* for 10 min and OD of the supernatant was measured. The difference of both values gave the OD of the sample. This procedure was necessary because OD values of the supernatant changed over the course of cultivation. For correlation between OD and cell dry weight (CDW), 5 mL samples were taken at the end of cultivation and transferred to dry, pre-weight 15 mL screwing cap reaction tubes. The tubes were centrifuged at 4816 ×*g* and 4°C for 15 min. The supernatant was discarded and pellets were washed two times with a 9 g L^-1^ NaCl solution. The tubes with the washed pellets were dried at 60°C for 48 h before they were weighed again and the BDM was calculated. Using the final OD of the cultures resulted in an average correlation coefficient of 0.3 g OD^-1^.

Fructose-containing samples were analyzed for fructose, acetic acid, and ethanol using enzymatic assays of Roche Yellow line (Hoffmann-La Roche, Switzerland). Samples from syngas grown cultures were analyzed for products using a 6890N GC (Agilent, United States) equipped with auto-sampler, ROTICAP-FFAP capillary column (0.5 μm, 30 m × 0.32 mm ID, Carl-Roth, Germany), and flame ionization detector. The carrier gas was helium with a pressure of 1 bar and a split ratio of 7.5:1. The analytical standard mixture consisted of 5 mM ethanol, 5 mM sodium acetate, and 9.09 mM isobutanol in 0.18 M HCl. Samples were prepared by acidifying 500 μL of the sample with 50 μL internal standard solution consisting of 100 mM isobutanol in 2 M HCl. Injection volume was 1 μL of sample or standard. The temperature profile of the column oven started with initial 60°C for 2 min followed by a temperature increase of 10°C min^-1^ up to an end temperature of 180°C. Total analysis time was 20 min. Due to incompatibility of available cyanide assays with the medium, analytics of cyanide content of samples could not be conducted.

## Results

### Experiments With Fructose as a Substrate

**FIGURE 2:**
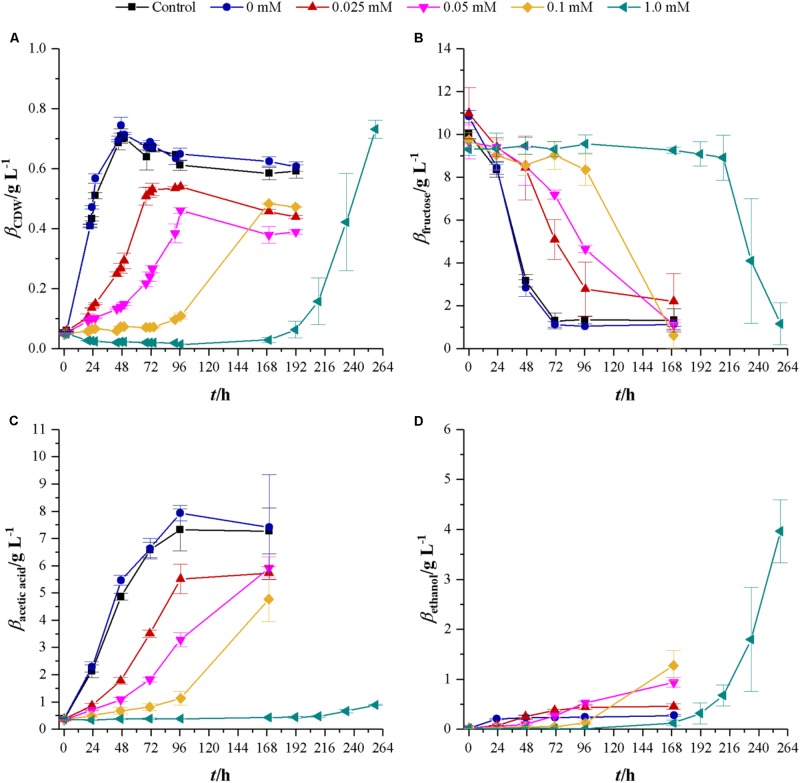
Response of fructose growing *C. ljungdahlii* to increasing concentrations of cyanide. Average values for three independent bottles per concentration. Control cultures (black squares) do not contain any phosphate buffer or cyanide while cultures with 0 mM cyanide (blue dots) contain 1 mL 100 mM potassium phosphate buffer at pH 11. Other cyanide concentrations are 0.025 (red triangles), 0.05 (magenta upturned triangles), 0.1 (yellow diamonds), and 1.0 mM (petrol tilted triangles). **(A)** CDW; **(B)** mass concentration of fructose; **(C)** mass concentration of acetic acid; and **(D)** mass concentration of ethanol. Average values of three independent cultivations.

First experiments on the influence of cyanide used concentrations of 0, 0.025, 0.05, 0.1, and 1.0 mM KCN in cultures with either fructose or synthesis gas as a substrate. The effects of cyanide on growth and product formation are shown in **Figure 2**. With increasing cyanide concentration, lag-phase increases and maximum growth rate decreases from 0.1 h^-1^ without cyanide to 0.08 (0.025 mM cyanide), 0.05 (0.05 mM cyanide), 0.02 (0.1 mM cyanide), and 0.05 h^-1^ (1.0 mM cyanide). Substrate consumption and product formation show this tendency as well. However, since the exponential growth phase in 0.1 mM cyanide happened between 96 and 168 h, where no samples were collected, it is likely that the maximum growth rate is closer to 0.05 h^-1^. While cultures without cyanide reach a maximum CDW of 0.73 ± 0.03 g L^-1^, all cyanide containing cultures reach, within the margin of standard deviation, the same maximum CDW of approximately 0.49 g L^-1^. The exception is the culture with 1.0 mM cyanide, where the maximum CDW is 0.73 ± 0.03 g L^-1^ the same as at cyanide-free conditions. At this cyanide concentration, cells do not start to grow until 168 h but from then on they consume the same amount of fructose as the other cultivations within the following 96 h. Another effect of increasing cyanide concentrations can be found with the formed products. *C. ljungdahlii* converts consumed fructose into acetic acid and ethanol. The yields for those two are 0.76 (acetic acid) and 0.03 g g^-1^ (ethanol) when growing without cyanide. Those yields shift with increasing cyanide concentrations. For acetic acid values of 0.61 (0.025 mM cyanide), 0.65 (0.05 mM cyanide), 0.48 (0.1 mM cyanide), and 0.06 g g^-1^ (1.0 mM cyanide) are obtained while the yields for ethanol are 0.05 (0.025 mM cyanide), 0.11 (0.05 mM cyanide), 0.14 (0.1 mM cyanide), and 0.48 g g^-1^ (1.0 mM cyanide).

**FIGURE 3:**
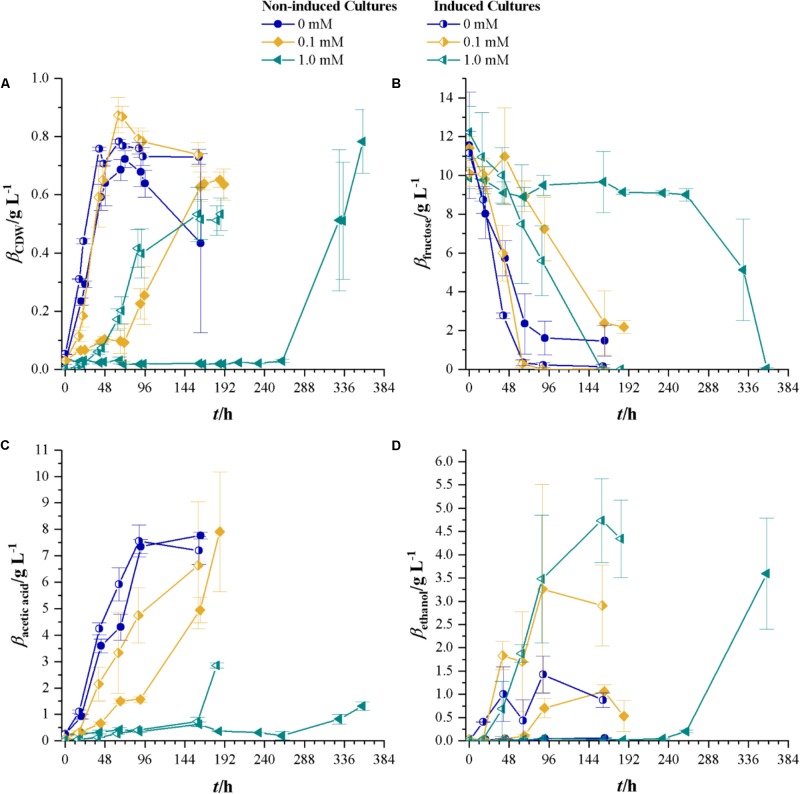
Non-induced *C. ljungdahlii* (full symbols) vs. the induced strain (half-filled symbols) at different cyanide concentrations with fructose as a carbon source. Cyanide concentrations are 0 (blue dots), 0.1 (yellow diamonds), and 1.0 mM (petrol tilted triangles). **(A)** CDW; **(B)** mass concentration of fructose; **(C)** mass concentration of acetic acid; and **(D)** mass concentration of ethanol. Average values of three independent cultivations. All experiments are conducted in triplicates.

Since *C. ljungdahlii*, once it starts growing at 1.0 mM cyanide, reaches CDW concentrations of the same value as without cyanide, the next step is to use the grown culture from 1.0 mM cyanide containing medium (aka induced strain) to inoculate fresh cyanide-containing medium. Therefore, experiments with 0, 0.1, and 1.0 mM cyanide were conducted using non-induced *C. ljungdahlii* and the induced strain. **Figure 3** shows the results of that comparison. Without cyanide present, the strains reach comparable maximum CDW of 0.72 ± 0.03 (non-induced strain) and 0.78 ± 0.11 g L^-1^ (induced strain). However, with increasing cyanide concentrations the induced strain shows no significant delay in growth at 0.1 mM cyanide and a lag-phase of about 24 h at 1.0 mM cyanide. The non-induced strain on the other side has a lag-phase of about 24 h at 0.1 mM cyanide and of about 260 h at 1.0 mM. Notably, the induced strain reaches a maximum CDW of 0.83 g L^-1^ at 0.1 mM cyanide, which is above the values found in cultures of both strains without cyanide. As for the maximum growth rates, the non-induced strain reaches values of 0.11 h^-1^ (0 mM cyanide), 0.05 h^-1^ (0.1 mM cyanide), and 0.04 h^-1^ (1.0 mM cyanide) while the induced strain reaches 0.10 (0 and 0.1 mM cyanide) and 0.06 h^-1^ (1.0 mM cyanide).

Substrate consumption of the induced and non-induced strains follows the same pattern as the growth at different cyanide concentrations. Fifty percent of fructose is consumed after 30, 48, and 96 h for the induced and 50, 120, and 324 h for the non-induced strain at 0, 0.1, and 1 mM cyanide, respectively. When looking at product formation, the induced and non-induced strains yield the same concentration of acetic acid at 0 mM cyanide and while the non-induced strain only produces 0.06 g L^-1^ ethanol at this cyanide concentration, the induced strain produces a maximum of 1.43 g L^-1^. For cyanide concentrations of 0.1 mM, the induced strain produces more ethanol than the non-induced strain. Maximum acetic acid concentrations in non-induced and induced strains cultures are comparable to the values found in cultures without cyanide. At the highest cyanide concentrations, both the non-induced and induced strains produce equal amounts of ethanol with the difference that the induced strain reaches the maximum concentration after 159 h while the non-induced strain takes 358 h. At 1.0 mM cyanide, the non-induced strain produces 1.3 ± 0.16 g L^-1^ acetic acid while the induced strain produces 2.85 ± 0.10 g L^-1^.

### Cultivations With Syngas as a Carbon and Energy Source

**FIGURE 4:**
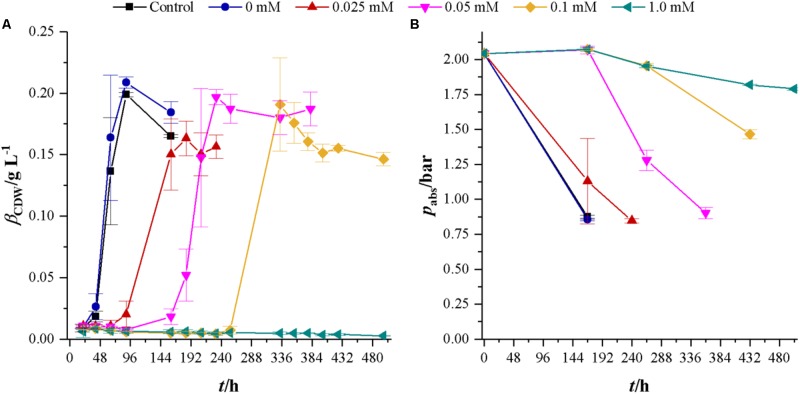
Development of CDW and headspace pressure of cultivations of *C. ljungdahlii* in the presence of different cyanide concentrations with syngas as a carbon and energy source. Each value is an average of three cultivations. Control cultures (black squares) do not contain any phosphate buffer or cyanide while cultures with 0 mM cyanide (blue dots) contain 1 mL of 100 mM potassium phosphate buffer at pH 11. Other cyanide concentrations are 0.025 (red triangles), 0.05 (magenta upturned triangles), 0.1 (yellow diamonds), and 1.0 mM (petrol tilted triangles). **(A)** CDW and **(B)** mass concentration of fructose. Average values of three independent cultivations.

After the effects of cyanide on heterotrophic growth on fructose were investigated, we studied the effects of cyanide on autotrophic metabolism of *C. ljungdahlii* at the same concentrations of cyanide as for heterotrophic growth. **Figure 4** shows the development of CDW and headspace pressure for cultures growing in the presence of 0, 0.025, 0.05, 0.1, and 1.0 mM KCN with a gas atmosphere of 21.1 ± 3.13 vol% H_2_, 23.1 ± 3.74 vol% CO, 10.4 ± 1.70 vol% CO_2_, and 39.5 ± 6.85 vol% N_2_. Cultures were seeded from 5% inoculated cultures grown for 7 days with syngas as carbon and energy source. The control culture (without phosphate buffer) and the culture with 0 mM cyanide show the same growth behavior. For cyanide concentrations of 0.025, 0.05, and 0.1 mM the lag-phase increases with increasing cyanide concentration. However, despite that, cultures at 0.05 and 0.1 mM cyanide reach the same maximum CDW of about 0.20 g L^-1^ while the 0.025 mM culture reaches with 0.16 ± 0.01 g L^-1^ a slightly lower value. Maximum growth rates are 0.08 h^-1^ (control), 0.08 h^-1^ (0 mM cyanide), 0.03 h (0.025 mM cyanide), 0.04 h^-1^ (0.05 mM cyanide), and 0.04 h^-1^ (0.1 mM cyanide). No growth can be observed within 497 h in the presence of 1.0 mM cyanide.

The development of headspace pressure, representing the available substrate, shows a similar trend as the CDW development. Final pressure in cyanide-free cultures is 0.86 ± 0.01 bar. At this pressure, the gas atmosphere consists only of carbon dioxide and nitrogen (data not shown). With their lag-phase reflecting time delay, cultures with 0.025 and 0.05 mM cyanide reach the same pressure as cyanide-free cultures. At 432 h (18 days), the 0.1 mM cyanide containing culture reaches a pressure of 0.90 ± 0.04 bar but has only completely consumed carbon monoxide. Hydrogen partial pressure in that culture after 18 days is 34.42 ± 3.31 mbar. The pressure drop in 1.0 mM cultures is due to liquid and gaseous samples taken from the bottles since no growth or product formation occurred (data not shown).

**FIGURE 5:**
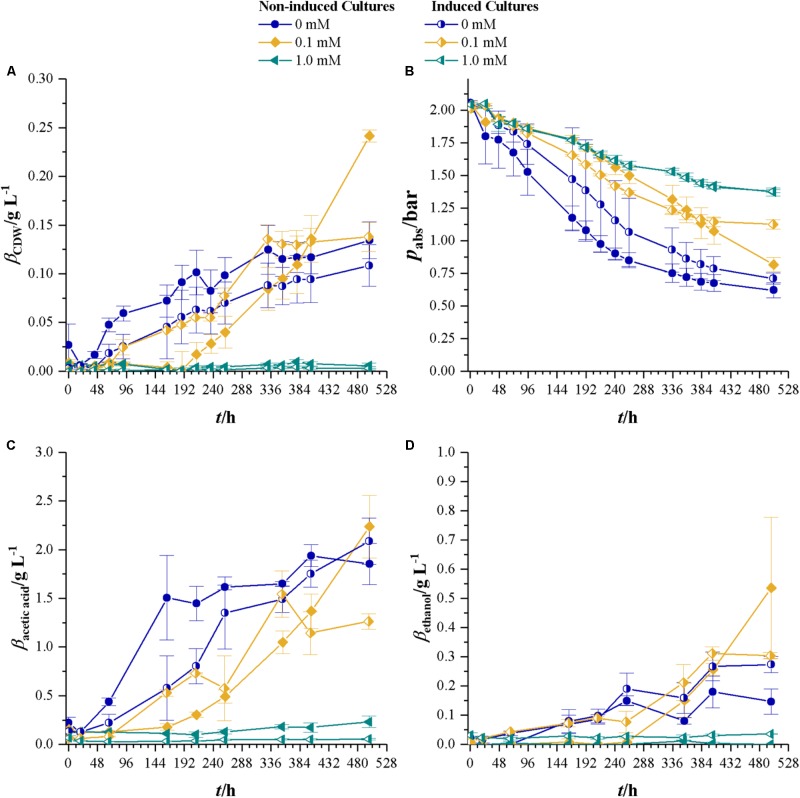
Non-induced *C. ljungdahlii* (full symbols) vs. the induced strain (half-filled symbols) at different cyanide concentrations with syngas as a carbon and energy source. Cyanide concentrations are 0 (blue dots), 0.1 (yellow diamonds), and 1.0 mM (petrol tilted triangles). **(A)** CDW; **(B)** mass concentration of fructose; **(C)** mass concentration of acetic acid; and **(D)** mass concentration of ethanol. Average values of three independent cultivations. Average data from triplicate experiments.

Similar to the approach with fructose growing cultures, bottles with 0, 0.1, and 1.0 mM were seeded with non-induced *C. ljungdahlii* and a strain induced on fructose in 1.0 mM cyanide-containing medium. Pre-cultures for this experiment were grown for 48 h with syngas as carbon and energy source. Syngas composition was 27.1 ± 0.6 vol% H_2_, 29.0 ± 0.5 vol% CO, 13.5 ± 0.2 vol% CO_2_, and 28.3 ± 0.4 vol% N_2_. **Figure 5** shows the results for CDW, headspace pressure, and concentrations of acetic acid and ethanol. Cultures of the non-induced strain without cyanide show the shortest lag-phase and reach maximum CDW of 0.12 ± 0.02 g L^-1^. Non-induced cultures at 0.1 mM cyanide do not start to grow before 188 h and reach a maximum CDW of 0.24 ± 0.01 g L^-1^. As for the induced cultures, the ones with 0 and 0.1 mM cyanide show the same growth pattern in the first 259 h. The cyanide-free cultures of induced *C. ljungdahlii* reach a maximum CDW of 0.11 ± 0.02 g L^-1^ while the ones at 0.1 mM cyanide reach 0.13 ± 0.01 g L^-1^. No increase in CDW is measured for 1.0 mM cyanide for both non-induced and induced strain. Maximum growth rates for non-induced cultures are 0.04 (0 mM cyanide) and 0.03 h^-1^ (0.1 mM) and for the induced strain 0.03 (0 mM cyanide) and 0.05 h^-1^ (0.1 mM cyanide). However, exponential growth could only be found during the first 48 h after growth started. Initial headspace pressure is 2.03 ± 0.01 bar. For each cyanide concentration, the non-induced cultures reach the lowest final pressure with the exception of 1.0 mM where no growth occurred.

Final acetic acid concentrations are within the same margin of standard deviation for non-induced and induced strain at 0 mM cyanide and the non-induced at 0.1 mM cyanide. On average they reach 2.06 g L^-1^. At 353 h maximum concentration of acetic acid in 0.1 mM cultures of the induced strain are measured with 1.54 ± 0.24 g L^-1^. Final ethanol concentrations are below 0.3 g L^-1^ for all cultures except the non-induced cultures in 0.1 mM cyanide containing medium which have 0.53 ± 0.24 g L^-1^ ethanol.

## Discussion

### Experiments With Fructose as a Substrate

For acetogenic bacteria cyanide is known to reversibly inhibit the enzyme CODH (Thauer et al., 1974; Ragsdale et al., 1983a,b; Terlesky et al., 1986; Grahame and Stadtman, 1987; Ha et al., 2007) but available literature deals only with the effect of cyanide on crude extract or isolated CODH from acetogens. **Table 1** summarizes the results of experiments under heterotrophic conditions. Our results show that for cyanide concentrations of 0.025–0.1 mM the resulting maximum CDW is lower in cyanide-free cultures but at 1 mM cyanide concentration it is within the same range as the control cultures. Although when comparing the non-induced with the induced strain at 1.0 mM cyanide in **Figure 3**, the latter grows to a maximum CDW lower than both strains without cyanide while the non-induced strain, after 261 h lag-phase, reaches a CDW comparable to the control cultures. The reason for this remains unknown. Although the lag-phase of the induced strain in the presence of 1.0 mM cyanide is prolonged compared to 0.1 mM, the cultures start to grow after 20 h. This is less than a 10th of the time the non-induced strain needs to start growing under these conditions, which shows that *C. ljungdahlii* can be conditioned to grow in the presence of cyanide. Once induced, growth happens at the same rate as without cyanide induction (0.1 h^-1^) up to CN^-^ concentrations of 0.1 mM.

**Table 1:** Results for CDW, growth rate, lag-phase, and product yield of heterotrophic cultures in the presence of different concentrations of cyanide.

Culture	β_CDW,max_/g L^-1^	μ_max_/h^-1^	*t*_lag-phase_/h	*Y*_P/S,aa_/g gL^-1^	*Y*_P/S,etOH_/g g^-1^
**Non-induced cultures**					
0 mM cyanide^∗^	0.73	0.10	0	0.76	0.03
0.025 mM cyanide	0.54	0.08	0	0.61	0.05
0.05 mM cyanide	0.46	0.05	0	0.65	0.11
0.1 mM cyanide^∗^	0.60	0.04	49	0.65	0.10
1.0 mM cyanide^∗^	0.75	0.05	227	0.09	0.42
**Induced cultures**					
0 mM cyanide	0.78	0.10	0	0.63	0.08
0.1 mM cyanide	0.87	0.10	0	0.65	0.28
1.0 mM cyanide	0.53	0.06	20	0.23	0.35


*^∗^Mean values from the initial experiments and the experiments where induced and non-induced strains are compared with each other; *Y*_*P/S,aa*_, yield of acetic acid per substrate and *Y*_*P/S,etOH*_, yield of ethanol per substrate.*

**FIGURE 6:**
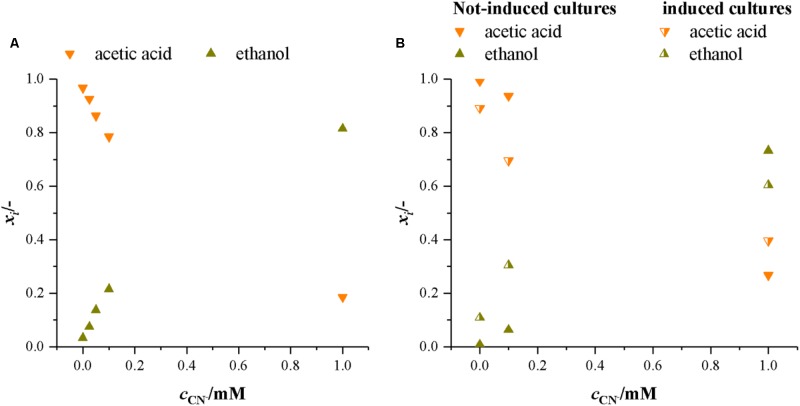
Molar ratio of products formed by *C. ljungdahlii* in the presence of increasing concentrations of cyanide with fructose as a carbon source. Average data from three different cultivations. **(A)** Experiments with non-induced cultures only. **(B)** Experiments with non-induced and induced cultures. Orange, upturned triangles, acetic acid and dark yellow triangles, ethanol.

At heterotrophic growth conditions, the purpose of the Wood–Ljungdahl pathway (WLP) is to recycle NAD^+^ and ferredoxin by capturing the CO_2_ from decarboxylation of pyruvate. CODH is the central enzyme in the WLP. If it is inactivated by cyanide, capturing of CO_2_ is no longer possible and the next possibility of recycling NAD^+^ and ferredoxin is by converting acetic acid to ethanol (Köpke et al., 2010). This shifts the main product from acetic acid to ethanol. **Figure 6** presents the molar ratio of products (*x_i_* = *c_i_* (Σ*c*_Products_)^-1^) in both sets of experiments with fructose as a carbon source. It is evident that with increasing cyanide concentration more ethanol is formed while the amount of acetic acid decreases. This indicates that with increasing cyanide concentration CODH activity is increasingly inhibited and NAD^+^ and ferredoxin are recycled by conversion of acetic acid to ethanol. The overall product yield is supporting this as well. With no CODH activity, the maximum possible product yield is 0.51 g g^-1^. In our experiments, the *Y*_P/S_ decreases from 0.80 ± 0.08 g g^-1^ at 0 mM cyanide to 0.55 g g^-1^ and 0.47 g g^-1^ in the non-induced cultures and 0.58 g g^-1^ in the induced cultures at 1.0 mM cyanide.

### Cultivations With Syngas as a Carbon and Energy Source

**FIGURE 7:**
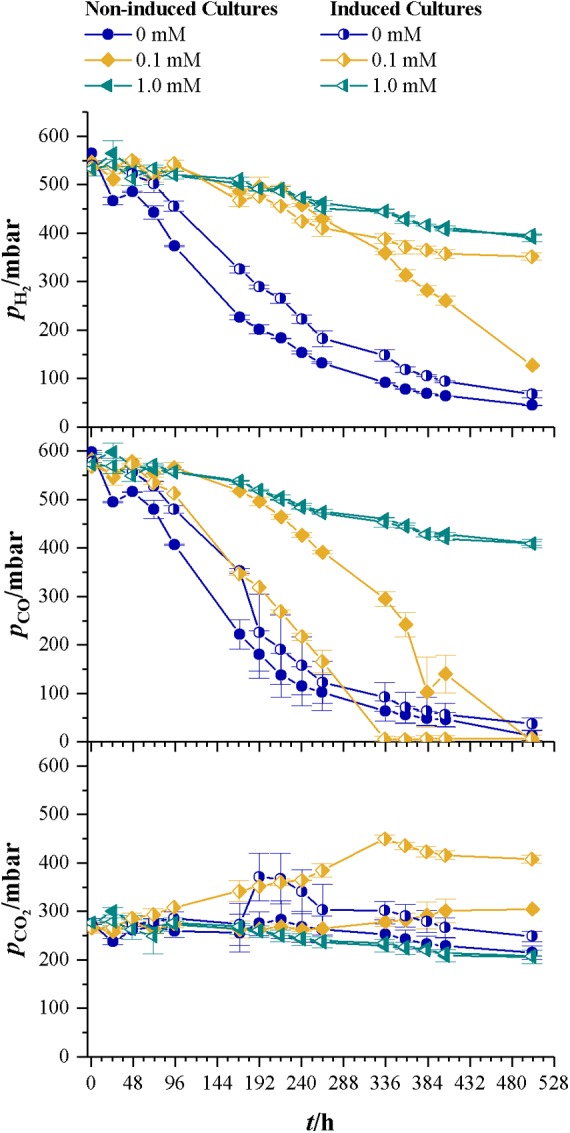
Development of partial pressures of H_2_, CO, and CO_2_ for non-induced *C. ljungdahlii* (full symbols) vs. the induced strain (half-filled symbols) at different cyanide concentrations with syngas as carbon and energy source. Cyanide concentrations are 0 (blue dots), 0.1 (yellow diamonds), and 1.0 mM (petrol tilted triangles). Since no growth occurred in the bottles with 1.0 mM cyanide, those values represent the pressure drop due to collection of liquid and gaseous samples. Average values of three cultivations per cyanide concentration.

**Table 2** summarizes the results for CDW, maximum growth rate, and lag-phase of the experiments under autotrophic conditions. Similar to the results with fructose as a carbon source, the lag-phase is prolonged with increasing levels of cyanide, but no increase in CDW or consumption of substrates is measurable at 1.0 mM cyanide. Even the induced strain does not grow at that concentration. 1.0 mM cyanide seems to be a concentration at which no autotrophic growth of *C. ljungdahlii* is possible. This result is in accordance with the findings from fructose grown cultures, where product spectrum and product yield indicate a loss of most of the CODH activity at 1.0 mM cyanide. Autotrophic growth on CO or CO_2_ is only possible with the WLP functional. Inhibitory effects of cyanide on CODH will directly result in reduced growth and product formation. However, up to 0.1 mM cyanide the final CDW and maximum growth rate seem unaffected by cyanide. **Figure 7** shows the development of the partial pressures of hydrogen, carbon monoxide, and carbon dioxide in the headspace of the culture bottles. At 0.1 mM cyanide, the CO consumption in the induced strain cultures starts after a lag-phase of 48 h and the non-induced strain after 192 h. Both show no other signs of cyanide inhibition when compared with the cyanide-free cultures but completely consume the available carbon monoxide. This indicates that either cyanide is actively degraded or the organism adapts to it in another way. Whatever the mechanism behind the adaption is, it seems to influence hydrogenase activity. Usually, in cultures growing on CO and H_2_, hydrogenases are inhibited by CO until the liquid CO concentration is low enough to revoke the inhibition (Vega et al., 1989). While for both strains hydrogen consumption starts at *p*_CO_ of 300 mbar, only about 50 mbar H_2_ are consumed by the induced strain (the difference between last measured partial pressure in 1.0 and 0.1 mM culture) whereas the non-induced strain consumes 260 mbar H_2_. Hydrogenases are usually not inhibited by cyanide (Adams et al., 1981) and since the non-induced strain does consume 260 mbar of hydrogen in the presence of 0.1 mM CN^-^, an inhibitory effect of cyanide on hydrogenase activity in the induced strain cultures seems unlikely.

**Table 2:** Results for CDW, growth rate, and lag-phase of autotrophic cultures in the presence of different concentrations of cyanide.

Culture	β_CDW,max_/g L^-1^	μ_max_/h^-1^	*t*_lag-phase_/h
**Non-induced cultures**			
0 mM cyanide^∗^	0.17	0.06	32
0.025 mM cyanide	0.16	0.03	65
0.05 mM cyanide	0.20	0.04	160
0.1 mM cyanide^∗^	0.21	0.04	221
1.0 mM cyanide^∗^	No growth	No growth	No growth
**Induced cultures**			
0 mM cyanide	0.11	0.07	43
0.1 mM cyanide	0.14	0.06	66
1.0 mM cyanide	No growth	No growth	No growth


*^∗^Mean values from the initial experiments and the experiments where induced and non-induced strains are compared with each other.*

How *C. ljungdahlii* is adapting to cyanide could not be identified during this study. Hydrocyanic acid in aqueous solution is known to slowly disintegrate into NH_3_ and formic acid (Roempp, 2012). While at first glance this could indicate that growth after a prolonged lag-phase is due to autocatalytic disintegration of cyanide, it is refuted by the fact that the induced strain shows only effects of inhibition when exposed to concentrations of 1 mM cyanide. Another possibility could be that *C. ljungdahlii* upregulates enzymes like cyanase, cyanidase, nitrilase, or rhodanese to neutralize HCN (Dubey and Holmes, 1995). However, none of these enzymes can be found in the published complete genome of *C. ljungdahlii* DSM 13528 (Köpke et al., 2010) and the product and yield data from heterotrophic cultures show no signs of cyanide degradation. Further investigation of the transcriptome and proteome at different cyanide concentrations is necessary to determine in what way *C. ljungdahlii* adapts to cyanide.

## Conclusion

The work presented here shows that *C. ljungdahlii* has prolonged lag-phases when grown in the presence of cyanide with fructose or syngas as carbon and energy source. But once the growth starts, the growth rate and maximum CDW are similar to those in cultures without cyanide. Even more, our results are the first to show that *C. ljungdahii* can be adapted to cyanide. Literature commonly states that syngas used as a substrate for fermentation has to be as clean as possible to avoid inhibitory effects (Xu et al., 2011; Daniell et al., 2012; Liew et al., 2016). This may be also achieved by varying gasifier conditions to minimize clean-up (Daniell et al., 2012). On the other hand, as has been described in the section “Introduction,” some impurities counter the effect of others and can even protect against severe inhibition when applied as a mixture. Unfortunately, the lack of studies using mixtures of impurities or crude syngas for the cultivation of whole cells makes it difficult to give a common statement to that topic.

Reported cyanide contents of crude syngas can reach values up to 2500 ppm (Broer et al., 2015) which would result in an equilibrium concentration of dissolved cyanide of 24.3 mM at 37°C. However, typical cyanide contents of crude syngas from wood or straw are below 25 ppm for wood (Boerrighter et al., 2013) and are about 250 ppm for straw (Kurkela et al., 1996) resulting in maximum liquid concentrations of 0.24 (wood) and 2.4 mM (straw) but in any case more than 247 L of gas per liter of medium is necessary to achieve that concentration. Assuming a gas feed rate of 0.1 vvm it would take more than 41 h of gas sparging to get to that concentration and the actual concentration the culture has to deal with when it is started to be fed with cyanide containing gas is much lower. Nevertheless, further investigation in bioreactors with a continuous feed of cyanide-containing gas is necessary to make a statement at which cyanide load the crude syngas gas needs to be purified prior to fermentation.

## Author Contributions

FO designing of cyanide inhibition experiments, supervision of experiments and evaluation of results, and writing of the manuscript. MZ designing of cyanide inhibition experiments and supervision of experiments, input on the evaluation of results, and critical revision of the manuscript. OO performance of experiments with non-induced strains. EH performance of comparison experiments with induced and non-induced strains. AN substantial input on experimental design and evaluation of results, and critical revision of the manuscript.

## Conflict of Interest Statement

The authors declare that the research was conducted in the absence of any commercial or financial relationships that could be construed as a potential conflict of interest.
